# The Yin and Yang of Immunity in Stem Cell Decision Guidance in Tissue Ecologies: An Infection Independent Perspective

**DOI:** 10.3389/fcell.2022.793694

**Published:** 2022-02-07

**Authors:** Vaishali Garg, Shashank Chandanala, M. David-Luther, M. Govind, Roshni Ravi Prasad, Anujith Kumar, S. Jyothi Prasanna

**Affiliations:** Manipal Institute of Regenerative Medicine, Manipal Academy of Higher Education (MAHE), Bangalore, India

**Keywords:** inflammation, stem cells, immune-stem cell cross talk, inflamm-aging, DAMPs, TLRs, mesenchymal stem cells, stem cell niches

## Abstract

The impact of immune system and inflammation on organ homeostasis and tissue stem cell niches in the absence of pathogen invasion has long remained a conundrum in the field of regenerative medicine. The paradoxical role of immune components in promoting tissue injury as well as resolving tissue damage has complicated therapeutic targeting of inflammation as a means to attain tissue homeostasis in degenerative disease contexts. This confound could be resolved by an integrated intricate assessment of cross-talk between inflammatory components and micro- and macro-environmental factors existing in tissues during health and disease. Prudent fate choice decisions of stem cells and their differentiated progeny are key to maintain tissue integrity and function. Stem cells have to exercise this fate choice in consultation with other tissue components. With this respect tissue immune components, danger/damage sensing molecules driving sterile inflammatory signaling cascades and barrier cells having immune-surveillance functions play pivotal roles in supervising stem cell decisions in their niches. Stem cells learn from their previous damage encounters, either endogenous or exogenous, or adapt to persistent micro-environmental changes to orchestrate their decisions. Thus understanding the communication networks between stem cells and immune system components is essential to comprehend stem cell decisions in endogenous tissue niches. Further the systemic interactions between tissue niches integrated through immune networks serve as patrolling systems to establish communication links and orchestrate micro-immune ecologies to better organismal response to injury and promote regeneration. Understanding these communication links is key to devise immune-centric regenerative therapies. Thus the present review is an integrated attempt to provide a unified purview of how inflammation and immune cells provide guidance to stem cells for tissue sculpting during development, organismal aging and tissue crisis based on the current knowledge in the field.

## Introduction

Immune response to injury determines the outcome of the healing process and efficient restoration of organ functions. Thus integrating inflammation and immune system targeted strategies to regenerative therapies is an interesting proposition. Knowledge of how immune system components and inflammatory signals interface with stem cells and progenitor cells in tissue niches is key to develop novel immune-centric regeneration therapies. An inverse relationship between efficient regeneration and immune competence exists as we go up the evolutionary ladder. In mammals as development proceeds injury responses proceed from being regenerative to reparative which resonates with the development of an intricate and complex immune system. A shift in scarless wound healing and regeneration in neonates to imperfect repair in adult mammals is mainly attributed to emergence of an intricate adaptive immune system through development. Primitive immune cells emerging from hematopoietic waves before definitive hematopoiesis have important developmental roles in shaping organogenesis, these cells later co-occupy organ specific niches along with stem cells and adapt to tissue specific functions. However the precise relevance of immune system and their roles in tissue regeneration and homeostasis are only being appreciated recently.

Contemporary advancement in technology to fate map cells and availability of precise tools to analyze tissue niches has renewed awareness on the role of immune system in tissue regeneration beyond its traditional role in pathogen immunity. Immune-mechanisms orchestrating repair and/or regeneration from tissue stem/progenitor cells depend on the 1) quality of immune resolution (scarring, fibrotic or scarless, chronic *versus* acute inflammation) 2) timing (embryonic/perinatal/adult/aged) 3) tissue of interface (immune privilege status) 4) complex niche specific micro-environmental factors (microbiota, nerve inputs *etc.*) and systemic components (infiltrating circulating immune cells into tissue micro-niches, metabolic stresses *etc.*). The present review evaluates current knowledge on how immune perturbations and innate immune functions impact stem cell fate decisions and how alarm signals from endogenous niche components including stem cells and their differentiated progeny impact immune-responses in tissue microenvironments in health, aging and disease.

## Alarmins as Sterile Inflammatory Triggers and Influencers of Stem Cell Decisions in Tissue Niches

Cellular damage, stress and death triggers an alarm response in tissue niches sending a warning signal to undamaged niche cells to equip themselves with necessary arsenal to protect themselves and maintain tissue integrity under crisis. Stem cells need to sense these alarms adequately to preserve themselves from exhaustion and orchestrate their differentiation hierarchies based on present tissue demands. Alarmins are immunomodulatory and activate endogenous- or recruit exogenous immune system components into tissues which supervise repair responses. Alarmins include damage associated molecular patterns or DAMPs together with pathogen associated molecular patterns (PAMPs). A vicious loop of DAMP signaling and sterile inflammation if unresolved can drive tissue degenerative processes impacting stem cell functions and regeneration. DAMPs include diverse molecules like uric acid, mtDNA, extracellular ATP, HSPs, amyloid β, S100, HMGB1, and ECM proteins (Bianchi, 2007).

**FIGURE 1:**
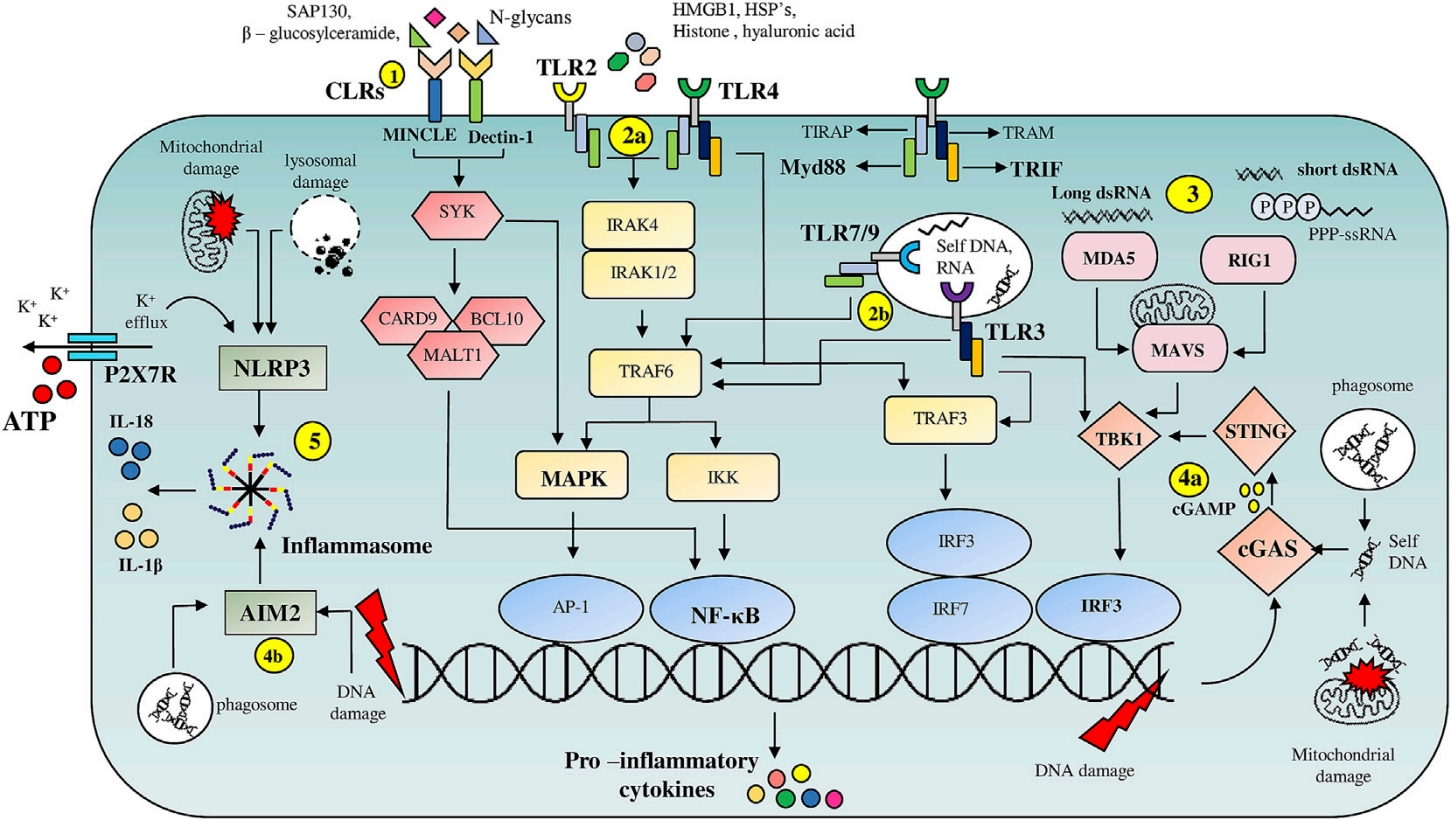
Overview of PRR mediated DAMP signaling pathways: **(1)** C-type lectin receptors (CLR’s), Macrophage inducible C-type lectin (MINCLE) and Dectin-1, on binding to DAMP’s such as Sin3A associated protein 130 (SAP-130), β—glucosylceramide and N-glycans respectively, trigger NF-κB and MAPK signaling pathways *via* engaging spleen tyrosine kinase (SYK). **(2)** Cell surface localized TLR’s **(2a)**, TLR2 and TLR4, are activated by intracellular proteins such as HMGB1, HSP’s, histones, whereas endosomal TLR’s **(2b)**, TLR7, TLR9 and TLR3 are activated by nucleic acids. TLR’s activate multiple pro-inflammatory signaling pathways through their adaptor proteins MyD88 and TRIF. **(3)** Host derived RNA molecules are recognized by RLR’s such as RIG-1 and MDA5 which then *via* mitochondrial antiviral signaling system (MAVS) adaptor protein activate TBK-1 resulting in IRF3 dependent transcription of pro-inflammatory cytokine genes. **(4)** cGAS and AIM2 serve as cytoplasmic nucleic acid sensors. cGAS **(4a)** binds to DNA fragments derived from either genomic damage or from debris phagocytosed after tissue damage resulting in the release of cyclin GMP—AMP (cGAMP) second messenger which on binding to stimulator of interferon genes (STING) drives IRF3 dependent transcription of inflammatory cytokine genes. **(4b)** AIM-2 on binding to nucleic acid fragments results in the assembly of inflammasome facilitating release of pro-inflammatory cytokines such as IL-1β and IL-18. **(5)** NLRP3 based inflammasome assembly is triggered by numerous events such as ATP-P2X7R mediated K^+^ efflux and mitochondrial or lysosomal damage resulting in the release of IL-1β and IL-18.

**FIGURE 2:**
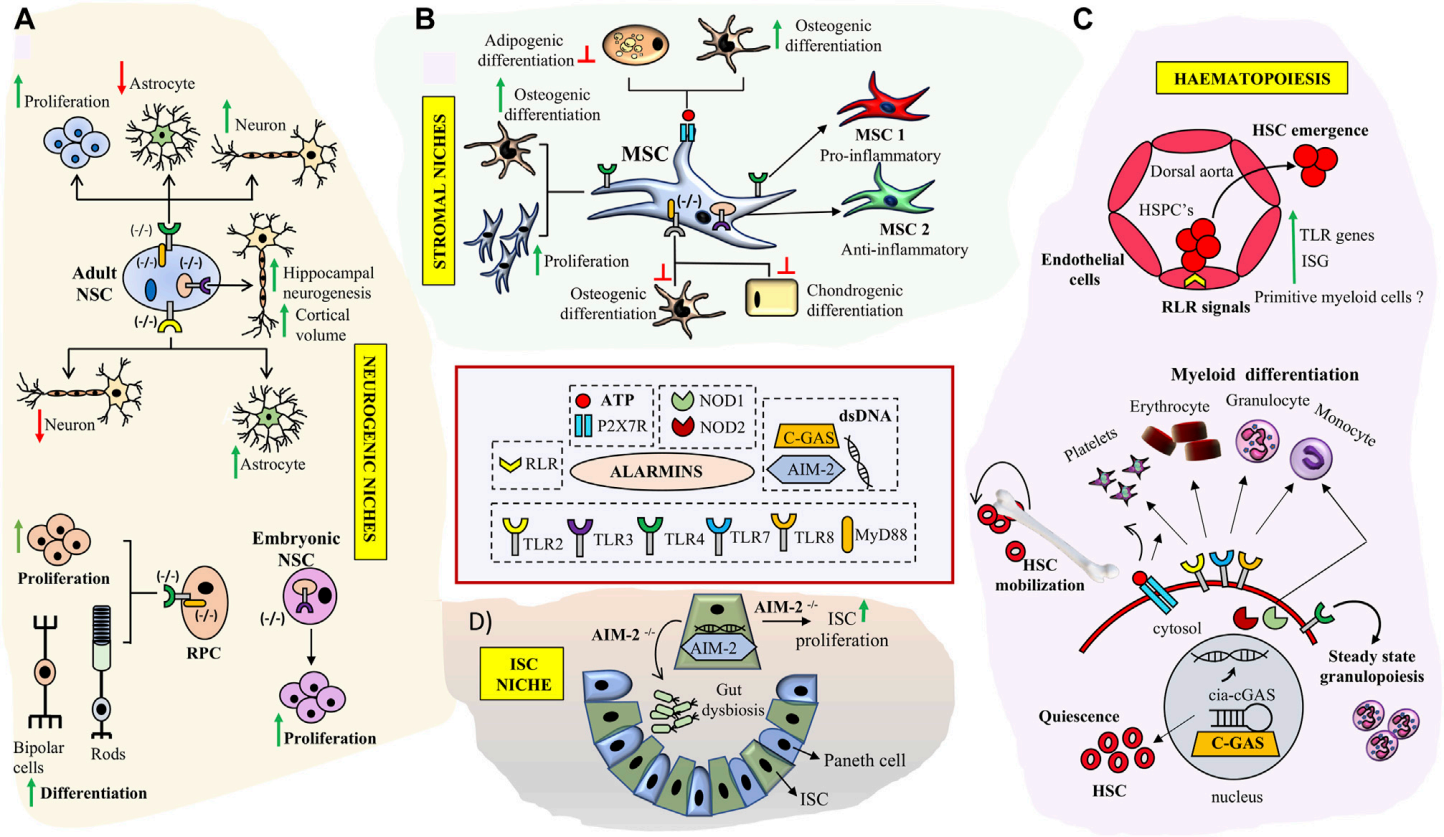
Role of key Alarmin signals in neuronal, hematopoietic, intestinal and stromal tissue niches: **(A)** TLR signaling deficiency impacts NSC proliferation and neurogenesis in SVZ, DG and enhances RPC proliferation and differentiation in retina. **(B)** Triggering specific TLR signaling pathways modulates immuno-plasticity and multi-lineage differentiation potential of MSCs and skews them into MSC1 (TLR4+) or MSC2 (TLR3+) phenotypes. Engagement of P2X7 receptor by ATP results in enhanced osteogenesis in MSCs **(C)** Broad roles of alarmin signals in HSC development and bone marrow niches. RLR signaling is implicated in HSC emergence through trans-differentiation of endothelial cell at HCCs during early development. TLR signaling and purinergic inputs impact HSC- quiescence, differentiation and mobilization respectively. Interaction of cGAS with cia-cGAS prevents HSC exhaustion. TLR signaling inputs are implicated in myeloid cell type differentiation. **(D)** Double stranded DNA sensor AIM2 prevents gut dysbiosis and inhibits ISC proliferation in ISC niche.

Evolutionarily conserved Pattern Recognition Receptor pathways (PRRs) recognizing altered patterns on microbes, viruses or endogenous danger signals lying at the helm of endogenous tissue surveillance mechanisms guide stem cell fate decisions and are key alarmin sensors (Figure 1). Out of the four major subfamilies of PRRs; TLRs (Toll like Receptors), NLRs (NOD like receptors), RLR (RIG-1like receptors) and CLRs (C-type Lectin receptors), TLRs are the most well studied (Figure 1). TLR signaling is implicated in stem cell fate guidance in neuronal, stromal, Hematopoietic and Intestinal niches (Figures 2A–D). Initial studies in *Drosophila* implicated developmental roles of TLRs in establishment of dorsal-ventral axis specification, synaptogenesis and axonal guidance apart from roles in anti-microbial defense (Anderson et al., 1985; Hashimoto et al., 1988; Halfon et al., 1995; Rose et al., 1997). Since then 10- mouse and 12- human functional TLRs are discovered. Surprisingly despite brain being an immune-privileged organ TLR expression has been noted in mammalian Central Nervous system (CNS) in the absence of damage or infection. Insights from TLR2-, TLR4-and MyD88-deficient (D) mice decisively indicated its role in adult neurogenesis. Both TLR2 and TLR4 seem to be exhibiting conflicting influences on proliferation/renewal and neuronal differentiation. TLR2D Neural stem cells (NSCs) exhibited reduced neuronal differentiation and enhanced astrocytic differentiation. However, MyD88-deficient mice exhibited enhanced proliferation as well as neuronal differentiation from NSCs similar to TLR4D mice indicating TLR family receptor specificity (Rolls et al., 2007). Balanced differentiation from NSCs to neurons versus astrocytes is critical to maintain brain homeostasis. Hence TLR related pathways are key rheostats in balancing renewal and neuronal differentiation choices (Figure 2A). Similar to its role in hippocampal neurogenesis TLR4 also impacts retinal progenitor cell (RPC) proliferation and neuronal differentiation during early post-natal periods. This effect was mimicked by RPCs deficient in common adaptors of TLR signaling, MyD88 and TICAM1 (Shechter et al., 2008). The cessation of RPC proliferation in adulthood coincides with elevated TLR4 levels in adult eye. Interestingly growth factor administration in TLR4D mice results in considerable resumption of RPC proliferation observed in early postnatal retinas suggesting that TLR4 could serve as an important gatekeeper and microenvironment sensor restricting adult RPC proliferation (Figure 2A). Endosomal TLR3 enhances the proliferative capacity of embryonic but not adult NPC (Lathia et al., 2008) and TLR3D mice exhibit enhanced neurogenesis (Okun et al., 2010). Since no definitive endogenous ligands to TLRs in the central nervous system and retinal cells have been proposed, it is possible that these molecules exist to sense deviations from homeostasis such as matrix degradation products and convey important environmental information to fine tune neurogenic decisions. Given that other TLR members are expressed in brain during the developmental window (Barak et al., 2014) it would be interesting to decipher whether TLRs apart from TLR-2, -4, and -3 have specific roles in neural fate choices.

Sterile inflammatory signaling seems to be evolutionarily conserved in regulating Hematopoietic stem cell (HSC) production during embryonic development. Studies on HSC emergence in zebrafish and mouse indicate that tonic innate immune cell signaling in the absence of microbial challenge can contribute to HSC orchestration and control of cell numbers much before the development of a functional adaptive immune system. Known IFNγ target genes have been shown to be expressed in human fetal HSCs. Intra-arterial Hematopoietic cluster cells (HCC) emerging from the hemogenic endothelium exhibited an innate immune gene signature with enrichment of TLR pathways and IFN target genes indicating existence of sterile immune signaling networks in both mouse and human fetal HSCs (Li et al., 2014). A combination of pro-inflammatory stimuli emerging from the primitive myeloid cells could serve to provide an inflammatory microenvironment which impacts vertebrate HSPC numbers and lymphoid progenitors. These results point towards a broader role for primitive myeloid cells in aiding HSPC production apart from their role in fighting pathogens. Since the early conceptus exists in a fairly aseptic microenvironment to induce inflammatory or innate immune circuits, prevalence of apoptotic and hypoxic niches could serve as possible TLR signaling triggers.

In addition to differentiated blood cells both mouse and human HSCs express most TLR family members and their downstream adaptor proteins (Figure 2C). Stimulation of human HSCs with TLR7/8 or TLR2 agonists results in myeloid differentiation. TLR2 locus is active in the earliest E7.5 EMPs (Erythroid-Myeloid progenitors) apart from E9.5 definitive HSCs during mouse development and the co-expression of c-Kit and TLR2 can be used to temporally track these waves. Further fate mapping experiments substantiated the contribution of c-Kit+/TLR2+ cells to adult hematopoiesis (Balounová et al., 2019). Apart from TLRs, NLRs, NOD1 and NOD2 are also expressed on human HSCs and engagement of these ligands results in differentiation towards the monocyte/macrophage lineages along with upregulation of intracellular defensins indicating that HSCs have mechanisms to protect themselves for microbial onslaughts without engaging an adaptive immune storm (Sioud and Fløisand, 2009). Recently, TLR4 has been ascribed as an indispensable sensor of steady state granulopoiesis from HSC independent of G-CSF (Granulocyte-Colony Stimulating Factor) inputs implying an interesting role of commensal gut microbiome in immune health (Marklin et al., 2020).

Apart from the role of TLR and basal inflammatory signaling in developmental hematopoiesis sterile inflammation has been shown to be triggered through RLRs *via* endogenous repetitive elements which are specifically expressed during endothelial to hematopoietic transitions during HSC emergence in zebrafish models (Figure 2C). The relevance of RLR signaling was further confirmed in mouse fetal liver HSCs wherein attenuation of RLR pathway mediators RIG-1, MDA-5 or TRAF6 reduced inflammatory signaling and impacted clonogenicity of HSCs upon *in vitro* serial passaging (Lefkopoulos et al., 2020). Interestingly stem cells also express intracellular dsDNA receptors such as cGAS (cyclic GMP-AMP synthase) and AIM (Absent In Melanoma2). These receptors constitute stem cell intrinsic machinery to sense crisis (Naik et al., 2018). cGAS and AIM bind double stranded DNA (dsDNA) either generated as an intermediate of a viral infection or through endogenous molecules generated during cell stress without any pathogen specific attributes. In HSCs, cGAS binds the circular RNA cia-GAS, regulating cGAS with high affinity and fine-tuning its sensitivity to endogenous dsDNA in turn limiting HSC exhaustion promiscuously (Figure 2C) (Xia et al., 2018). The importance of cytoplasmic DNA sensing is exaggerated in ISC (Intestinal stem cell) niche where loss of AIM results in gut dysbiosis and uncontrolled ISC proliferation (Figure 2D) thus enhancing susceptibility to colon tumors (Man et al., 2015).

MSCs (Mesenchymal stem cells) are integral constituents of Hematopoietic niches and express a wide range of TLR receptors. Minor variations in TLR expression patterns are noted based on species and their tissue of origin (Shirjang et al., 2016). Triggering of TLRs on MSCs by endogenous/exogenous DAMPs or PAMPs could serve as alarms to use their immune-modulatory arsenal, adjust stromal proliferation/migration responses and differentiation abilities thus aiding establishment of tissue homeostasis. Immune-plasticity and migration of MSCs are modified on specific TLR-agonist engagement and based on it two polarized MSC states analogous to polarized monocytes, MSC1 (pro-inflammatory) and MSC 2 (anti-inflammatory) have been proposed. Specifically TLR-3 priming enhances their immune dampening effects whereas TLR-4 priming establishes a pro-inflammatory molecular signature (Waterman et al., 2010) (Figure 2B). Stimulation of mouse MSCs with TLR2 ligand inhibits induced mesodermal differentiation potential along with reduction in migratory potential. Supporting this observation MSCs derived from MYD88−/− mice failed to differentiate in to osteocytes and chondrocytes whereas adipogenic differentiation was retained (Pevsner-Fischer et al., 2007). In consensus, TLR4 stimulation of human MSCs resulted in an increase in proliferation and osteogenic differentiation by engaging Wnt3a and Wnt5a signaling (He et al., 2016). Further, IL-1R/MyD88 signaling attenuates bone regeneration in a mouse model of calvarial defect. Though most TLR KO (knock out) mice exhibit normal bone regeneration MyD88^−/−^ and IL-1R1^−/−^ mice exhibit faster bone regeneration in injured microenvironments. Exogenous administration of MyD88^−/−^ MSCs at the site of bone defect accelerated mineralization and bone regenerative responses. Further engineering the regenerative niche by fibrin matrix functionalization using IL-1R1/MyD88 inhibitors improved bone depositions and repair of the bone defect (Martino et al., 2016). Thus TLRs and their downstream signaling components could help fine tune MSC- migratory, proliferatory, immune-modulatory and differentiation abilities based on tissue requirements (Figure 2B). Aberrant niches such as that existent in tumors could impact responsiveness of MSCs to TLRs. MSCs from multiple myeloma (MM) patients’ exhibited similar differentiation abilities in response to TLRs as that of control subjects on *ex vivo* culture. However, MM MSCs exhibited stable alterations in secretion of IL8 and failed to activate crucial pathway such as ERK1/2 upon TLR2 stimulation which impacted their responsiveness to growth factors such as EGF (Pevsner-Fischer et al., 2012). The inability of MSCs to respond to PAMPs in tumor microenvironments could be crucial in stromal remodeling and tumor evolution. In addition to TLRs, intracellular NLRs have also been shown to be expressed in different MSCs and activation of NLRs with synthetic agonists seems to impact their differentiation potential (Kim et al., 2010). However the relevance of NOD receptors in Mesenchymal differentiation choices *in vivo* is still elusive.

Most DAMPs signals through PRRs but extracellular nucleotides above certain threshold levels are indicative of cell stress and exert their effects *via* purinergic cell surface receptors. Autocrine eATP is noted from several cells and eATP levels peak during cellular stress. eATP is an evolutionarily conserved DAMP from mammals, fish, insects and plants. In mammals, eATP is known to bind to cell surface P2 receptors expressed on many tissues throughout the body. P2 receptors are categorized into two major classes: ligand-gated ion channels P2X1-7 receptors and metabotropic G protein-coupled P2Y receptors (Burnstock and Knight, 2004) Although functions of different P2 receptors have been studied in different stem cells types, P2X7 by far has been the most widely studied of them all. Especially in the hematopoietic niche, Purinergic signaling is an important component bridging sterile inflammation to HSC activation. ATP sensitive P2 receptors modulate multiple aspects of HSC biology including quiescence proliferation, senescence, differentiation and migration (Figure 2C). P2YR (high affinity receptors) sense low ATP levels whereas P2XR (low affinity receptors) seems to curb the response when inflammation peaks forming an auto-regulatory loop. P2XR receptor is known to activate the inflammasome resulting in release of IL-1β (Ratajczak and Kucia 2020). The resolution of this loop is mediated by action of ecto-nucleotidase enzymes which convert pro-inflammatory ATP to immune-suppressive Adenosine. HSC’s derived from P2Y14^−/−^ animals failed to restore hematopoiesis when transplanted in irradiated mice (Cho et al., 2014). Adenosine generating ecto-enzyme CD73^−/−^ mice exhibit enhanced inflammation (Zernecke et al., 2006). ATP signaling reduces quiescent HSCs along with CMP and GMP progenitor numbers. However an increase in mature myeloid cells was noted in the bone marrow (Rossi et al., 2012). Thus, ATP can act as “find-me” signals from stressed cells and enhance the myeloid response to clear debris and rescue functionality (Elliot et al., 2009). ATP release in the circulation can potentiate HSC egress from the marrow niche facilitating patrolling of peripheral tissues for a crisis. Eventually a demand based differentiation can be triggered at the site of tissue crisis with HSCs and their progeny supervising the tissue repair process. Apart from HSCs, bone marrow Mesenchymal stem cells expressing purinergic receptors also co-ordinate responses to eATP. Bone marrow MSC’s isolated from osteoporotic post-menopausal women had inherently reduced propensity to differentiate towards the osteogenic lineage which was rescued by activation of P2X7 (Noronha-Matos et al., 2014). Ovariectomized mice treated with P2X7 agonist bzATP showed improved bone parameters and reduced marrow adiposity implicating the role of ATP sensing receptors in modulating osteo-adipo balance for bone marrow mesenchymal progenitor cells (Figure 2B) (Li et al., 2015). In an animal model of inflammation mediated osteoporosis the wild type mice showed reduction in bone mineral density, bone strength and trabecular microarchitecture when compared to P2X7R KO mice clearly demonstrating the role of P2X7R in inflammation induced bone loss (Kvist et al., 2015). Thus P2 receptors integrate inflammation induced signals and fine-tune differentiation decisions in response to inflammation.

Both autocrine as well as paracrine secretion of ATP impacts neural stem cell renewal, migration and differentiation (Lin et al., 2007; Grim and Zimmermann, 2010). Thus the absolute concentration of ATP is carefully controlled by NTDPase enzymes whose concerted actions results in generation of Adenosine. Neural progenitors express P2Y receptors and short range purinergic signaling through these receptors impact ventricular zone NPCs cell expansion. Further ATP cleaving NTDPases are selectively localized in the neurogenic sub-ventricular zones and dentate gyrus where neuronal differentiation proceeds from NSCs indicating that a tight control of ATP induced purinergic signaling is exercised in neurogenic niches (Lin et al., 2007). Further the impact of P2Y1 on adult neurogenesis seems to depend on growth factor availability (Mishra et al., 2006). In the early neurogenic window P2X7 receptor expressed on neuroblasts contribute to clearance of apoptotic cells by activating innate phagocytosis (Lovelace et al., 2015). During a pathological inflammatory insult or ischemia, a spike in eATP activates P2X7 receptors expressed on almost all central nervous system cells and if unchecked by NTDPase activity can lead to excitotoxic cell death and triggers a sequelae of neuro-inflammatory reactions in microglial cells. Thus, depending on the ATP/Adenosine ratio traumatic stroke induced events can either lead to neuronal/glial cell death or could lead to neuroprotection (Burnstock 2015) and can impact NSC renewal and differentiation decisions during traumatic injury (Cavaliere et al., 2015).

Taken together understanding alarmin regulatory mechanisms could give a handle to detect early tissue defects and develop timely intervention strategies to handle progressive degenerative diseases.

## Resident Immune Cells in Tissue Microenvironments: Roles in Maintenance of Tissue Integrity and Stem Cell Differentiation

**FIGURE 3:**
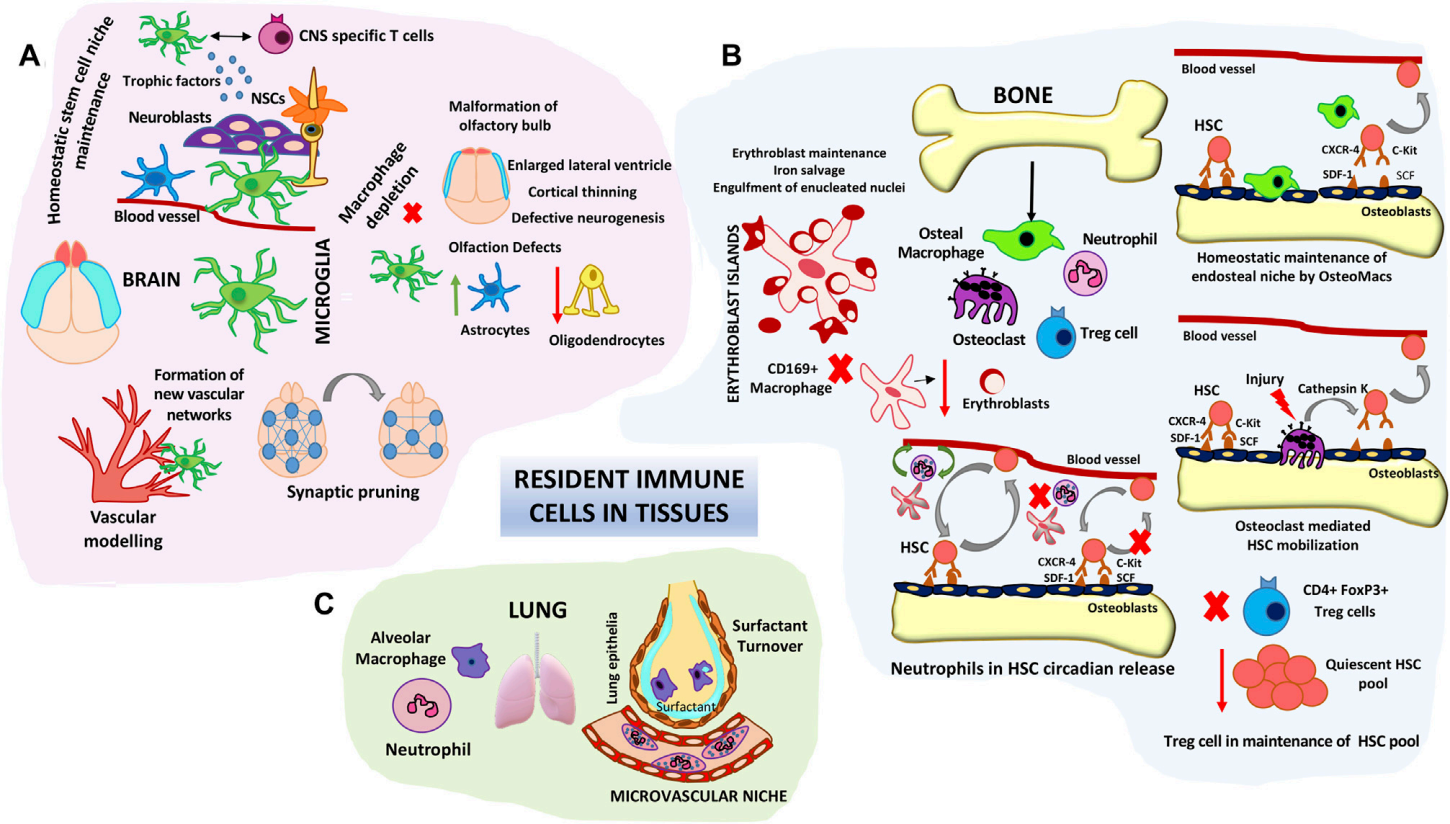
Roles of resident immune cells in tissue homeostasis and stem cell maintenance/differentiation. **(A)** Microglial cell functions in brain tissue homeostasis. Schematic represents the roles of microglia in fine tuning neuronal synapses, maintaining the neural stem niche and facilitating vascular modelling. Microglial depletion studies have shown structural defects in different areas of the brain such as the olfactory bulbs, cortex and lateral ventricles. **(B)** Resident Immune components in HSC niche homeostasis. Bone macrophages (Osteomacs) as well as osteoclasts modulate endosteal niches and influence HSC retention and egress into circulation. Central CD169 + macrophages form specialized erythroblast support niches in the bone marrow. Intimate cross-talk between neutrophils and stromal macrophages control the steady state circadian trafficking of HSCs. Immune-regulatory FoxP3^+^ T_reg_ cells prevent exhaustion of endosteal HSC. **(C)** Alveolar macrophages and neutrophils comprise key lung niche components. Vessel associated neutrophils are key barriers to pathogenic invaders and environmental alarms. Alveolar macrophages are involved in lung surfactant clearance.

There is growing evidence which suggests that innate immune cells are not just inflammation facilitators and adaptive immunity helpers but have housekeeping homeostatic tissue functions. Innate immune system components are native in most organs. Amongst them the macrophages are widespread components in most tissues and are characterized by tissue specific phenotypes and functions. Their functions range from apoptotic body clearance, removal of cellular byproducts, maintenance of endothelial barrier functions and metabolic homeostasis apart from the canonical immune surveillance functions (Davies et al., 2013; Theret M et al., 2019). Evidence from fate mapping studies and single cell RNA sequencing analysis indicate that a part of tissue macrophage heterogeneity arises from their developmental origin (Bian et al., 2020). Based on these studies tissue resident macrophages have been categorized as yolk sac derived, fetal liver derived and infiltrating bone marrow derived (Wu and Karen, 2021). Microglial cells in the brain seem to originate mainly from the earliest primitive yolk sac wave (around E8.5–9). With the exclusion of the microglia, where the formation of blood-brain barrier isolates them from other circulating precursors, tissue resident macrophages can be constituted by precursors from overlapping hematopoietic waves during development and self-renew in local tissue environments during the life span. In most tissues following a damage inducing crisis, macrophages can renew from local pools as well as be replenished by circulating blood progenitors which reposition themselves in tissue niches (Theret et al., 2019). However microglial restoration seems to happen through tissue specific yolk sac progenitors with minimal contribution from peripheral cells as long as the blood-brain barrier (BBB) is intact (Huang et al., 2018). Macrophage depletion studies with Csf1^op^/Csf1^op^ mice, Csf1r^−/−^/Csf1^−/−^ mice or using phagocytic cell depleting agents such as clodronate indicate the importance of these populations in organ development, their cross talk with stem cells in tissue niches and highlight their importance in maintaining organ homeostasis (Dai et al., 2002; Erblich et al., 2011; Winkler et al., 2010). As a prototypical example microglial depletion during development results in defects in vessel sprouting and synaptic pruning impacting neuronal connectivity and the brain vascular niches (Figure 3A). Further polarized microglial states could induced neurotoxicity or facilitate neurogenesis in a context dependent manner in adult brain (Tay et al., 2017). Microglial populations are intimately associated with NSCs, NPCs, migrating Neuroblasts and vasculature and can extend processes to communicate with ventricle lumen under physiological conditions. Depletion of microglia by intra-ventricular administration of CD11b-SAP are detrimental to subependymal NSCs implicating their proactive impact on adult neurogenesis (Sirerol-Piquer et al., 2019). Surprisingly in SCID (Severe Combined Immuno-deficient) mice hippocampal neurogenesis was severely impaired which could not be rescued by microenvironment enrichment indicating that apart from macrophages other immune cells such as CNS-specific T cells could also impact adult neurogenesis (Figure 3A). Further a close co-operation between microglia and CNS-specific T cells is needed to mediate their effects on neurogenesis (Ziv et al., 2006). Apart from tissue specific functions, as a paradigm, in all tissue niches engulfment of effete tissue cells by macrophages through efferocytosis are critical to dampen unwanted inflammation.

A prototypical example of innate cells influencing stem cell decisions is that of bone marrow hematopoiesis. This is not surprising given the fact that HSC differentiation is driven by the need of an ever replenishing and dynamic immune system. Two key innate immune players influencing the hematopoietic niches are neutrophils and macrophages (Figure 3B) (Casanova-Acebes et al., 2014). Trophic endosteal niche associated macrophages (Osteomacs) play important roles in retaining HSC at the endosteal niche surface. Depletion of bone marrow macrophages either through clodronate mediated approaches or by Granulocyte Macrophage Colony Stimulating Factor (GM-CSF) mediated loss of Osteomacs from the endosteal surface resulted in egress of HSCs into the blood stream by disruption of endosteal niche homeostasis (Winkler et al., 2010). Depletion of multiple heterogeneous pools of macrophages either through Csf1-r suicide gene targeting or specific depletion of marrow stromal associated CD169 + macrophage pools caused similar egress of HSCs into the blood (Winkler et al., 2010; Chow et al., 2011). Specialized bone remodeling osteoclasts belonging to the monocyte-macrophage lineage secrete cytokines like IL-8, MMPs and proteolytic enzymes such as cathepsin and aid in HSC mobilization majorly during stress as well as steady state (Figure 3B). Osteoclast secreted enzymes cleave endosteal niche components aiding HSC egress (Kollet et al., 2006). Macrophage islands in the bone marrow provide specialized micro-niches for erythroblast maturation (Figure 3B). Central macrophages in the erythroblast islands serve as nursing cells providing trophic support and are also involved in erythroblast enucleation (Yeo et al., 2019). Depletion of CD169-macrophages and not general monocytic populations resulted in reduced number of erythroblast islands without overt anemic complications under homeostatic condition implicating the role of these island associated macrophages in erythroid progenitor pool support in the bone marrow (Chow A et al., 2013). 90% of the neutrophils reside in the bone marrow and only a minor portion comprises the circulating pool during steady state. Depletion of endogenous neutrophils results in abrogation of circadian release of HSPCs into blood. Depletion of macrophages abolishes the ability of neutrophils to modulate HSPC circadian trafficking (Casanova-Acebes et al., 2013). These observations indicate a close co-ordination between neutrophils and macrophages (Figure 3B), probably by the phagocytic ability of macrophages to engulf effete neutrophils in influencing hematopoietic niches. Since Neutrophils are very sensitive to damage patterns and can change their migratory trajectories, changes in their circulatory conduits could impact hematopoietic niches as a feedback mechanism. Apart from the patrolling functions of neutrophils tissue resident neutrophils were noted to be residing in pulmonary capillaries and form an immune niche to immediately detect blood pathogens and decimate them and also protect vasculature during the process (Yipp et al., 2017; Granton E et al., 2018). Depletion of Alveolar macrophages by intra-tracheal administration of macrophage toxin resulted in increase in lung surfactant pool sizes indicating their specialized function in maintaining lung function (Forbes et al., 2007). In comparison to macrophage dominant tissue niches lung innate immune niche is predominated by neutrophils (Figure 3C). Analogous to this role recently phagocytic populations including neutrophils and macrophages were detected in sensory olfactory organ of zebra fish and both phagocytic population play important roles in lifelong neurogenesis of olfactory epithelia (Palominos and Kathleen, 2021). Though macrophages have longer residence times in tissues, neutrophils also have been shown to co-adapt to tissue microenvironments and cater to tissue specific functions and support organ homeostasis highlighting their non-canonical roles beyond infection immunity. In fact it is now appreciated that neutrophils have specific support function in healthy tissues too (Casanova-Acebes et al., 2018). Single cell RNA seq analysis of neutrophils from divergent tissues indicated unique tissue specific molecular signatures. Parabiosis experiments further confirmed that specification of neutrophil signatures were tissue acquired and imprinted, indicating neutrophil functional plasticity adapting to tissue demands (Ballesteros et al., 2020).

To date cells belonging to the mononuclear phagocytic system had been shown to occupy tissue niches and adaptive lymphocytes were found in the blood and lymphoid organs. However this traditional outlook had been questioned with the detection of innate-like lymphocytes in peripheral tissues which could have been positioned in peripheral tissues during peri-natal periods. In contrast to adaptive lymphocytes Innate Lymphoid cells (ILCs) lack antigen-specific receptors but nonetheless exhibit a broad functional spectrum like adaptive T cells and analogous to the T helper subsets are named as ILC1, ILC2 and ILC3. Despite presence of ILCs in SCID mice as well as immune-deficient humans the conventional immune responses to infection are severely compromised underscoring their role in microbial immunity. Data from mice studies indicate that developmentally they originate from a common innate lymphoid precursor (CILP) which do not possess T and B cell differentiation abilities and then finally infiltrate to different tissue *in utero*. Unlike most primitive tissue resident macrophages, ILCs arise during lymphoid progenitor orchestration (E 13.5 in mice) of fetal liver hematopoiesis. Further functional diversification in tissues to ILC-1, -2 or -3 is guided by signals as diverse as microbiota, dietary lipids and integrated tissue specific inputs during the perinatal period. However their migratory routes during development and during tissue injury in the adult is still elusive. Coincidently ILC2s co-inhabitate stromal tissue niches and were shown to modify epithelial barriers and affect tissue health. These cells could oscillate between different tonic functional states brought in to play by the functional needs of the tissue when challenged by microbial as well as physiological stresses and can thus function as immune tissue alarms (Kotas and Locksley, 2018). Based on these observations it is possible that early patterning of the ILC states could impact immune-stromal networks and later influence stem cell behavior in tissue niches. Molecular imprinting of these cells in tissue niches programmed by tissue specific growth factors, cytokines and signals could shape their tissue specific functions during renewal in tissues (Cortez and Marco, 2016; Zeis et al., 2020). Addressing specific roles of these cells would require precise fate mapping and depletion studies still missing in the literature to date.

Taken together evidences from development and tissue regenerative responses during crisis indicate that innate immune tissue niches are sculpted dynamically throughout development and lifetime to orchestrate tissue response and stem cell behavior during tissue homeostasis and imbalances. The broad immune-privilege at many somatic stem cell niches such as testis, hair follicle also indicates an active mechanism to keep immunity at bay. Apart from innate immune cells adaptive immune cells such as Treg cells have garnered attention for their role in influencing stem cell quiescence and differentiation in various stem cell niches. In fact stem cell niches such as the bone marrow, intestine, skin and hair follicle niches are shown to possess Treg cell reservoirs. Depletion of regulatory T cells subsets (T_reg_ cells) resulted in reduction in HSC pool (Figure 3B) (Fujisaki et al., 2011) as well as reduction in ISCs (Biton et al., 2018). During each hair cycle T_reg_ cell no fluctuates with high Treg numbers associated with the telogen phase when the stem cells are quiescent (Ali et al., 2017). Tissue resident T_reg_ cells secrete proactive molecules such as IL-10 and adenosine which impart an immune-suppressive milieu, reduce oxidative stress and prevent exhaustion of stem cell pools. During tissue perturbations a tight control of the inflammatory response can be exerted through co-operative interactions between Treg and resident macrophages and other innate immune cells such as ISCs to shift the gear towards a better tissue resolution by purposing their immune and tissue regenerative functions as per tissue demand. Analysis of transcriptional heterogeneity of T_reg_ cells isolated from immune and non-immune niches indicated that apart from enrichment of innate immune pathways and secretome, tissue T_regs_ are marked with a shared stem-cell molecular signatures indicative of a rather intimate link imprinted through a strong stem cell niche interaction (Zhang et al., 2020). Detailed roles of both T_reg_ cells and macrophages of many other niches apart from what is described above is reviewed by Naik et al. (2018).

In totality several immune cells form immune micro-niches within tissues during development and undergo dynamic restructuring during adulthood to maintain tissue homeostasis by supervising stem cell decisions.

## Non-traditional Roles of Complement in Tissue Remodeling/Regeneration and Stem Cell Decisions

Traditionally the complement pathway had been considered the soluble arm of the immune system orchestrating both innate and adaptive responses. Complement cascades are activated mainly in response to systemic crisis such as a pathogen insult or altered-self cells triggering inflammation and ultimately opsonisation and immune clearance. Complement is activated in a context dependent manner by either the classical pathway which engages the C1 antigen-antibody complex, the lectin pathway which involves recognition of pathogen and altered self-cell surfaces through mannose-binding lectin (MBL) or the low-grade constitutively activated alternative pathway which begins with the activation of C3. However, all these three pathways merge at C3 which is subsequently converted to C3a, an anaphylatoxin, and C3b, an opsonin. Further activation of C3 components depends on context specific versatile recognition molecules and receptors on cell and pathogen surfaces such as MBL, C1q and Ficolins. Despite low activation of the alternative pathway as a part of immune-surveillance, surface of healthy cells have complement regulators which prevent further nucleation (comprehensively reviewed by Merle et al. (2015).

Over the past 2 decades multiple studies have indicated role of complement and their receptors in organ development and maintenance of tissue homeostasis beyond their traditional roles in immune system. Complement proteins, their receptors and regulators are implicated in diverse physiological processes ranging from sperm-oocyte interaction, placental development, synaptic pruning, HSPC trafficking, proliferation and migration of cardiac progenitor cells and liver regeneration (Reviewed by Mödinger et al., 2018). In fact, complement is an evolutionarily conserved pathway across phylogenetically divergent species (Mastellos et al., 2013). A proactive role of complement proteins such as C3 and C5 is well studied in urodele models of limb and lens regeneration (Kimura et al., 2003). In embryonic chick retina complement fragment C3a induced complete regeneration from eye stem/progenitor cells (Haynes et al., 2013). The specific roles of complement proteins in regenerative tissues such as blastema and wound epithelium of urodeles as well as chicks furthered research on the role of complement in other animal models.

Classical Complement protein C1q, is involved in synaptic tagging for elimination by microglial cells both during brain development as well as in healthy adult hippocampus (Soteros et al., 2021). Disruption of complement components such as C1q, C3, and CR3 in microglial cells resulted in synaptic connectivity deficits (Stevens et al., 2007; Schafer et al., 2012; Wang et al., 2020). Further C1q-receptor interactions are reported to be involved in influencing human NSC migration, proliferation, and lineage commitment (Benavente et al., 2020). Blockade of C1q and C3a transiently altered hNSC migration and reversed astroglial fate changes after spinal cord injury (Hooshmand et al., 2017) indicating *in vivo* relevance of complement in neuronal regeneration and repair.

In bone biology C1s has been proposed to be involved in cartilage-bone transformation during endo-chondral ossification and several complement proteins were reported to be expressed in a spatio-temporal manner in the growth plates during bone development (Andrades et al., 1996). In fact complement receptors C3aR and C5aR, complement C3 and C5, and membrane-bound regulatory proteins CD46, CD55, and CD59 are expressed in most cell types (human MSC, osteoblasts, and osteoclasts) involved in bone remodeling. Complement proteins have been shown to be directly involved in osteoclastogenesis and migration of bone precursor cells (Ignatius et al., 2011). In line with these studies C3^−/−^ and C5^−/−^ mice exhibited impaired fracture repair (Ehrnthaller et al., 2013).

Studies from animal models of partial hepatectomy and acute hepatotoxicity have implicated an important role for Complement proteins in the liver regenerative processes (Mastellos et al., 2001; Clark et al., 2008; DeAngelis et al., 2012; Cresci et al., 2014). C3a/C3aR and C5a/C5aR1 were implicated to prime hepatocytes to re-enter mitotic phase (Strey et al., 2003; Min et al., 2016), regenerate hepatic mass and re-establish homeostasis.

Several studies have decisively implicated the role of complement in HSPC trafficking. HSPCs express functional C3aR and signaling through this receptor sensitizes their cross talk with the SDF-CXCR4 signaling axis influencing their homing and bone marrow retention potential (Reca et al., 2003; Ratajczak et al., 2004; Reca et al., 2006). Further the abrogation of diurnal release of HSPCs in C5^−/−^ mice emphasizes the dominant role of Complement cascades in HSPC trafficking circuits (Borkowska et al., 2016). Additionally, release of DAMPs such as ATP and ROS results in MBL pathway activation, C5 cleavage and HSC egress from bone marrow compartment under stress (Adamiak et al., 2017, reviewed by; Ratajczak et al. (2018), Cymer et al. (2020).

Interestingly it has been speculated that complement pathways can pose a barrier for teratoma formation from ESCs and iPSCs. ESC formed tumors more quickly in C3 deficient mice than wild type controls. Differentiated derivatives were more resistant to complement implicating that complement system could provide a tumor protection mechanism to the developing fetus during early embryogenesis (Koch et al., 2006). Further C5a receptors C5aR and C5L2 have been detected on human iPSCs and hESCs and stimulation of these receptors promoted maintenance of pluripotency in absence of FGF2 (Hawksworth et al., 2014). These studies shed light on an unexplored role of complement cascades in shaping early embryogenesis even before development of a fully functional immune system.

Thus, given the emerging role of complement in organ regeneration and its influence on stem cell behavior in several niches developing complement modulation strategies could be a parallel regenerative approach.

## Inflamm-Aging in Stem Cell Decisions and Regeneration in Tissues

Aging is associated with chronic low grade systemic inflammation concomitant with inefficient tissue regeneration and wound resolution. A three-way miscommunication between stem cells, stromal cells and immune cells is induced due to disruptors of these communication networks with age. Studies from aging hematopoietic niches have shed light on this aspect in an integrated manner. An aged hematopoietic niche is characterized by 1) decreased ability of HSCs for lymphopoiesis, myeloid biased differentiation, decrease in quiescence and increase in HSC pools 2) mesenchymal progenitor cell in the niche are biased towards adipogenesis (Singh et al., 2016) and exhibit a Senescence associated secretary phenotype (SASP) which further reiterates inflammatory signals 3) Impaired vascular functions and in-efficient phagocytic and scavenging function of marrow resident macrophage/myeloid lineage cells results in accumulation of senescent cells.

**FIGURE 4:**
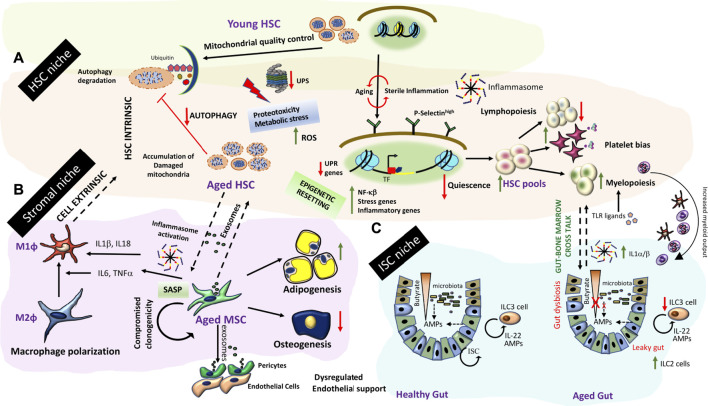
Inflamm-aging and tissue niche homeostasis **(A)** Inflamm-aging of the bone marrow niche involves HSC intrinsic aging mechanisms (dysregulation of protein degradation, accumulation of effete organelles and broad epigenetic resetting) concomitant with HSC exhaustion and a predominant myeloid bias further driven by other niche dependent mechanisms **(B)** Stromal components such as MSCs exhibit a SASP. Secretome from aged MSCs/stromal components drives polarization to pro-inflammatory macrophages, and result in changes in HSC pools. Further stromal progenitor cells are more adipogenic and do not adequately support endothelial functions resulting in bone marrow endothelial and stromal niche alterations affecting HSC output. **(C)** Gut-bone marrow cross talk in inflamm-aging. Aging is associated with a leaky gut causing gut dysbiosis and an iterative inflammation sequelae impacting ISC renewal and exposure of TLR ligands in the circulation. An enhanced demand for myeloid output along with inflammasome generated IL-1α/β exaggerates myeloid biased differentiation from HSCs in the bone marrow.

Both cell intrinsic and cell extrinsic mechanisms have been proposed for inflamm-aging in the bone marrow HSC compartment (Figure 4A). Transcriptome from aged HSCs (from mice maintained in pathogen-free conditions) is enriched in genes associated with inflammation, stress response and protein aggregation while chromatin remodeling and genome integrity associated genes were downregulated suggesting an epigenetic resetting with age. Of particular interest is upregulation of P-selectin and NFκβ activation, which is a master regulator of inflammatory gene expression (Chambers et al., 2007; Svendsen et al., 2021). Transplantation of P-Selectin^high^ HSCs from aged mice into irradiated young niches resulted in skewed myeloid differentiation from these cells. Further P-selectin ^high^ aged HSCs exhibited higher cycling (Svendsen et al., 2021). However, a direct role of P-selectin signaling in the aging phenotype has not been established. It is possible that P-selectin upregulation can impact the HSC migratory routes as well as leukocyte egress from bone marrow niches due to enhanced extravasation and further influence their homing and engraftment potential with age. Aged HSCs depicted strong platelet gene priming and myeloid bias alongside increases in HSC pools (Grover et al., 2016). These evidences are corroborated by age associated hematological pathologies which include decrease in bone marrow cellularity, higher incidence of anemia and decreased adaptive immunity in humans. Some of these age related changes could be HSC cell intrinsic driven by broad epigenetic resetting of the HSC genome. Transplantation studies old human HSCs into younger bone marrow niches indicated engraftment defects and inefficient generation of lymphoid progeny. Young HSCs engrafted better and even differentiated into different HSC lineages substantiating HSC intrinsic aging mechanism at least partly (Pang et al., 2011).

Apart from broad genome wide changes, aged HSCs exhibit high ROS (Reactive Oxygen Species) levels concomitant with metabolic/protein stress. These events are driven by breach of organelle quality control checks and loss of proteostasis which exaggerates intrinsic aging of quiescent HSCs (Figure 4A). Disruption/malfunctioning of both the UPR (Unfolded Protein Response) and Ubiquitin-Proteasome System (UPS) is responsible for dysregulated proteostasis. Further loss in autophagy processes adds to organelle-, metabolic- and protein-stresses in aged HSCs. HSCs from Vav-ATG7^−/−^ (conditional deletion of autophagy only in the HSCs) mice are incapable of bone marrow reconstitution post-irradiation exaggerating the importance of autophagy processes in maintenance of HSC integrity (Mortensen et al., 2011). It is still elusive whether metabolic stress results from increase in cycling of HSCs and if both processes are coupled together with age related changes in HSCs.

Bone marrow being a very dynamic niche, stem cells in this niche are subjected to various acute as well as chronic stresses through the life span of the organism. Most niche cells including HSCs express TLR family members which trigger signaling to damage and low-grade infections setting up an iterative inflammatory tone. In fact, an indication of this intrinsic immunity is imprinted in HSCs as depicted by a robust ISG (Interferon stimulated gene) signature in the absence of a prevailing infection. Interestingly, HSCs are unresponsive to Interferon due to high thresholds of ISGs. Surprisingly, differentiated derivatives of HSC have a narrow spectrum of ISGs but acquire IFN responsiveness indicating an adaptation of bone marrow microenvironment to protect exhaustion of LT-HSCs but at the same time engage progenitor responsivity to viral challenges (Wu X et al., 2018). Endogenous ligands in the absence of infection (eg. Alarmins derived through damaged organelles and their in-efficient degradation with age) can trigger TLR responses and sterile inflammation through inflammasome activation. In this context, NOD-like receptor NLRP3 inflammasome is particularly important in engaging upstream sterile triggers such as eATP, ROS, S1P, HMG-B1 and complement cleavage products to HSC egress and mobilization in both HSPC cell-autonomous and bone marrow micro-environment dependent manner (Extensively reviewed by Ratajczak and Kucia (2021). Aged HSCs in particular exhibit NLRP3 activation due to reduced Sirt2 and accumulated mitochondrial stress. Inactivation of NLRP3 or targeting the Sirt2-NLRP3-Caspase1 axis restored regenerative abilities of aged HSCs (Luo et al., 2019). Importantly, NLRP3 seems to couple metabolic, DAMP and complement triggers to HSPC trafficking (Ratajczak et al., 2018; Lenkiewicz et al., 2019; Ratajczak and Kucia, 2021; Thapa et al., 2021; Adamiak et al., 2020b) and production (Frame et al., 2020). Nlrp3 deficiency in the bone marrow microenvironment of the recipient mice as well as transplantation of Nlrp3^−/−^ precursor cell into wild type mice impaired HSPC homing and engraftment efficiencies. Interestingly, it has been proposed that NLRP3 regulates HSC migration by facilitating incorporation of CXCR4 into membrane lipid rafts (Adamiak M et al., 2020a). Thus, NLRP3 activation is emerging as a central point integrating upstream and downstream signaling inputs to modulate HSC behavior. Since NLRP3 inflammasome activation in HSCs as well as other innate cells in the bone marrow is integral to bone marrow aging and myelodysplastic syndromes, targeting NLRP3 is an interesting possibility to reverse inflamm-aging and promote homing and engraftment in transplant settings.

Some interesting evidence in the literature indicate inflammatory memory imprinted in the HSC genome due to a previous encounter which is passed on to their differentiated progeny. BCG induced a sustained reprogramming of HSCs and epigenetic modifications were passed on to macrophages/myeloid progeny which induced more protective immunity to virulent mycobacterial infection than naïve macrophages (Kaufman et al., 2018 Cell). Similarly western diet induced epigenetic reprogramming of GMP progenitors results in an inflammasome-mediated trained immunity precipitating in exaggerated systemic inflammation (Christ et al., 2018). Thus, even sterile inflammatory triggers from the niche can train bone marrow hematopoietic progenitors to respond differently to future threats impacting stem cell aging.

Aged Mesenchymal progenitor cells in the bone marrow as well as other tissues exhibit a senescent phenotype marked by loss of clonogenicity and breakdown of DNA repair responses. Exosomes secreted by senescent stromal cells communicate aberrant instructions to stem cells altering their decisions (Gnani et al., 2019 Aging Cell). Further, aged MSCs have altered immune-modulatory properties which impacts their intimate cross talk with marrow macrophages polarizing them towards an inflammatory phenotype (Figure 4B). Exposure of young HSCs to secretome of old MSCs induced inflammatory gene programs in HSCs (Gnani et al., 2019). In an isolated co-culture system of aged senescent endothelial cells and bone marrow MSCs, MSCs were shown to exhibit a pro-inflammatory phenotype with secretion of IL-6 and TNFα and stemness associated transcription factors (Lazzarini et al., 2019). Taken together aging related events set up a self-perpetuating sterile inflammatory loop in bone marrow microenvironment which impacts HSC function and immune homeostasis with age. In addition, bone marrow inflamm-aging sets up a chronic low grade systemic inflammatory tone which results in peripheral tissue niche dysfunction as well.

Skeletal dysfunction has been attributed to inflamm-aging in muscle stem cell niches. An increase in JAK/STAT3 signaling and inflammatory gene signatures had been noted in satellite cells from aged mice. Inhibition of STAT3 signaling in old satellite cells improved their engraftment abilities, symmetric expansion, and improved muscle regeneration *in vivo* (Price et al., 2014). Increased levels of inflammatory cells were noted in aged tissue which could have triggered the JAK/STAT3 signaling. The proactive role of systemic inflammation in muscle stem cell aging was proven in a recent study where parabiosis was performed between NFκβ^−/−^ mice which exhibited chronic systemic inflammation with a young mouse. Exposure of inflammatory factors through shared circulation resulted in an aged skeletal phenotype and dysfunction of skeletal stem/progenitor cells (Josephson et al., 2021) indicating the role of systemic inflammation in muscle aging phenotype. Aging is a key risk factor for myocardial diseases even in the absence of specific tissue damage or a persistent infection. Apart from resident M2 phenotype macrophages which are a constituent part of the cardiac niche, a substantial proportion of other leukocytes were found in the parenchyma of myocardium at steady state in young mice. Aged mice depicted change in leukocyte subsets together with functional and structural impairment (cardiac hypertrophy, fibrosis) of the myocardium along with spontaneous heart directed autoimmunity. Transfer of juvenile lymphocyte deficient Rag KO mice with heart draining mediastinal Lymph node (LN) cells from old mice indicated increased frequency of T cells in the myocardial niche concomitant with an aging phenotype. Importantly a skewing towards an IFNγ-producing Foxp3^-^ CD4^+^ population was noted in aged myocardial tissue indicating breakdown of peripheral tolerance mechanisms with age. These changes concomitant with accelerated senescence and inability of phagocytic cells to clear senescent cells effectively triggered myocardial degeneration. Further the cardio-tropism of the mediastinal LN cells in contrast to LN cells from other regions indicates the specific role of these lymphocyte subsets in the myocardial niche aging (Ramos et al., 2017).

Aging related factors could influence epithelial barrier integrity and functions in diverse niches. Of particular significance is the gut barrier dysfunction with age (Figure 4C). Gut barrier dysfunction can lead to gut dysbiosis or vice versa. Gut Epithelial barrier function is gated by tight junction physical barriers and chemical barriers of antimicrobial peptides (AMPs) synthesized by gut epithelial and immune cells. AMPs create microbial ecologies within intestinal niches and prevent them from invading gut epithelial cells. AMP production is regulated by intestinal cytokine production which is altered in advanced age. Further a mutual symbiotic relationship evolves between gut microbiota and the immune system around the perinatal period. Microbial stimulation during this developmental window shapes mucosal immunity. During aging a shift in the gut immune populations had been noted. A skewing of ILCs towards the ILC2 subset (mouse) and a decrease in ILC3 numbers (human) was noted. Thus, a two way communication between intestinal niche cells and gut microbiota is altered as a result of aging related factors. Through organismal life span, usage of antibiotics, glycemic stresses, metabolic adaptations etc. can change stability of microbial ecologies and impact microbial diversities. Oral pathogenic bacteria are kept in check by AMPs secreted by commensals and host cells in a collaborative manner. This collaboration is altered by sterile inflammation and loss of epithelial barrier function with age resulting in disruption of this feed forward regulatory network. Metabolites produced by these commensal bacteria from dietary components such as Short-chain Fatty Acids (SCFAs) e.g., butyrate are essential for maintenance of gut integrity. Butyrate has been shown to facilitate tight junction assembly and is a primary fuel for colonocytes. Exhaustion of these commensals results in a loss of barrier function and altered differentiation hierarchies from stem cells due to loss of these chemical gradients in the gut lumen (Figure 4C). A leaky gut exposes intestinal niche to previously unexposed bacterial triggers instigating TLR signaling and perpetuating intestinal inflammation. An increase in MAMPs (Microbe associate molecular patterns) in a leaky gut triggers PRRs on innate immune cells of different tissues causing perturbation in tissue homeostatic mechanisms (Walrath et al., 2021). A very recent study established the role of leaky gut derived microbes in triggering local TLR responses which enhance systemic levels of inflammatory cytokines like IL1α/β. IL1α/β enhances myeloid outputs from HSC, dampening overall immune fitness. Aged ILR1 KO mice or germ free mice maintained balanced lymphoid-myeloid differentiation potential like young mice indicating the importance of gut health and gut-bone marrow cross-talk in maintenance of balanced HSC renewal and differentiation (Kovtonyuk et al., 2021).

Thus systemic interactions between aging organ systems and inflamm-aging in the central bone marrow niche together sustains a chronic inflammatory loop affecting stem cell and progenitor cell behavior in local tissue niches and disturbing tissue-specific homeostatic networks at the organismal level with age.

## Inflammation Triggered Trans-Differentiation Events

**FIGURE 5:**
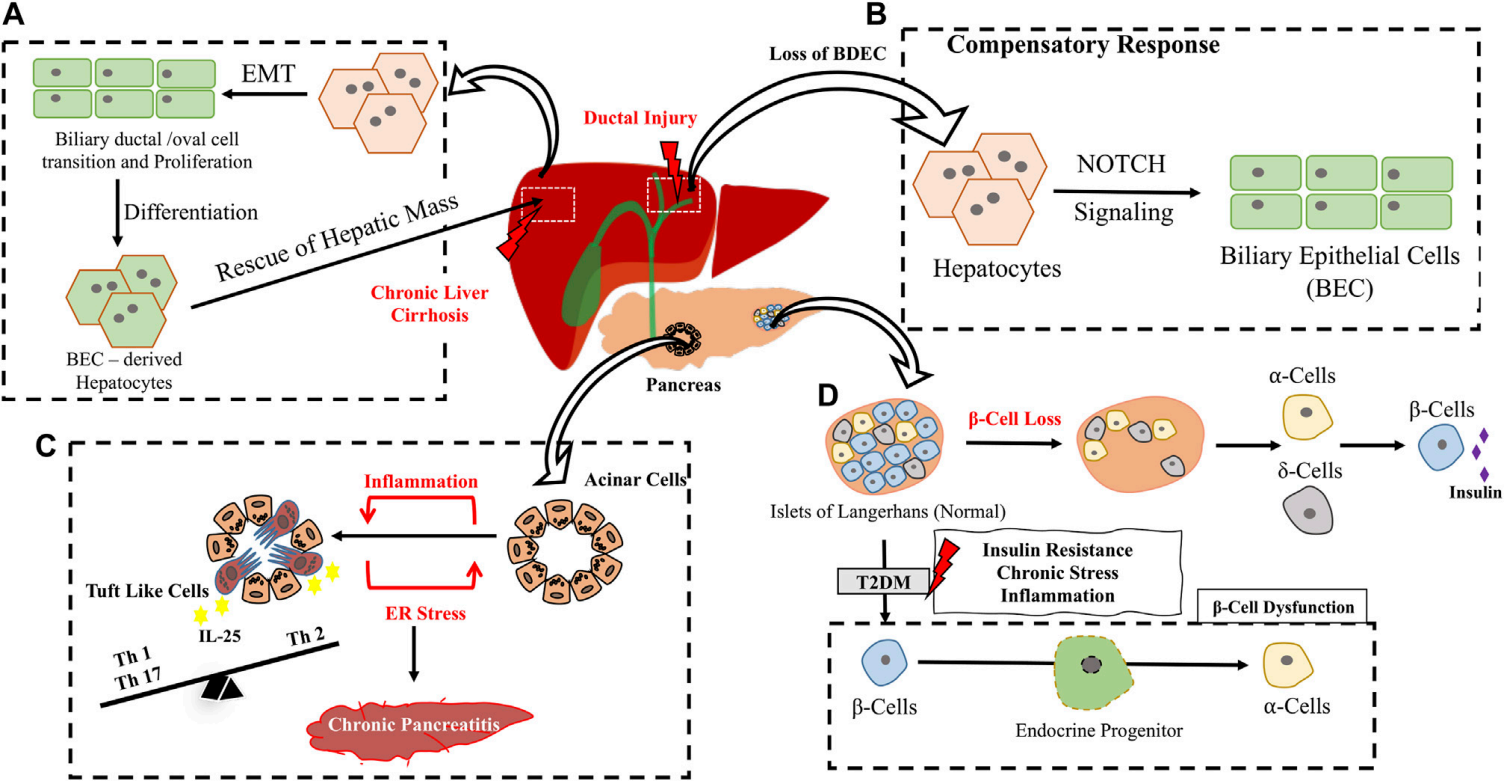
Inflammation/Injury induced trans-differentiation in Hepato-pancreatic system. **(A)** Under chronic liver cirrhosis/inflammatory conditions, normal hepatocytes from the adjacent region undergoes Epithelial to Mesenchymal Transition (EMT) followed by expansion through a ductal cell transition. BEC derived Hepatocytes replenish hepatic mass. **(B)** Compensation of BEC loss in chronic ductal injury through a hepatocyte to BEC trans-differentiation involving Notch signaling **(C)** Few acinar cells in the pancreas directly trams-differentiate to Tuft like Cells upon Chronic Pancreatitis shaping adaptive immunity in the niche. **(D)** Loss of β cells results in conversion of ⍺ and δ cells to β cell type to compensate for insulin requirement. Chronic stress prevalent in T2DM, leads to β cell dysfunction which includes loss of β cell integrity and conversion to ⍺-cells through an intermediate endocrine progenitor transition.

**FIGURE 6:**
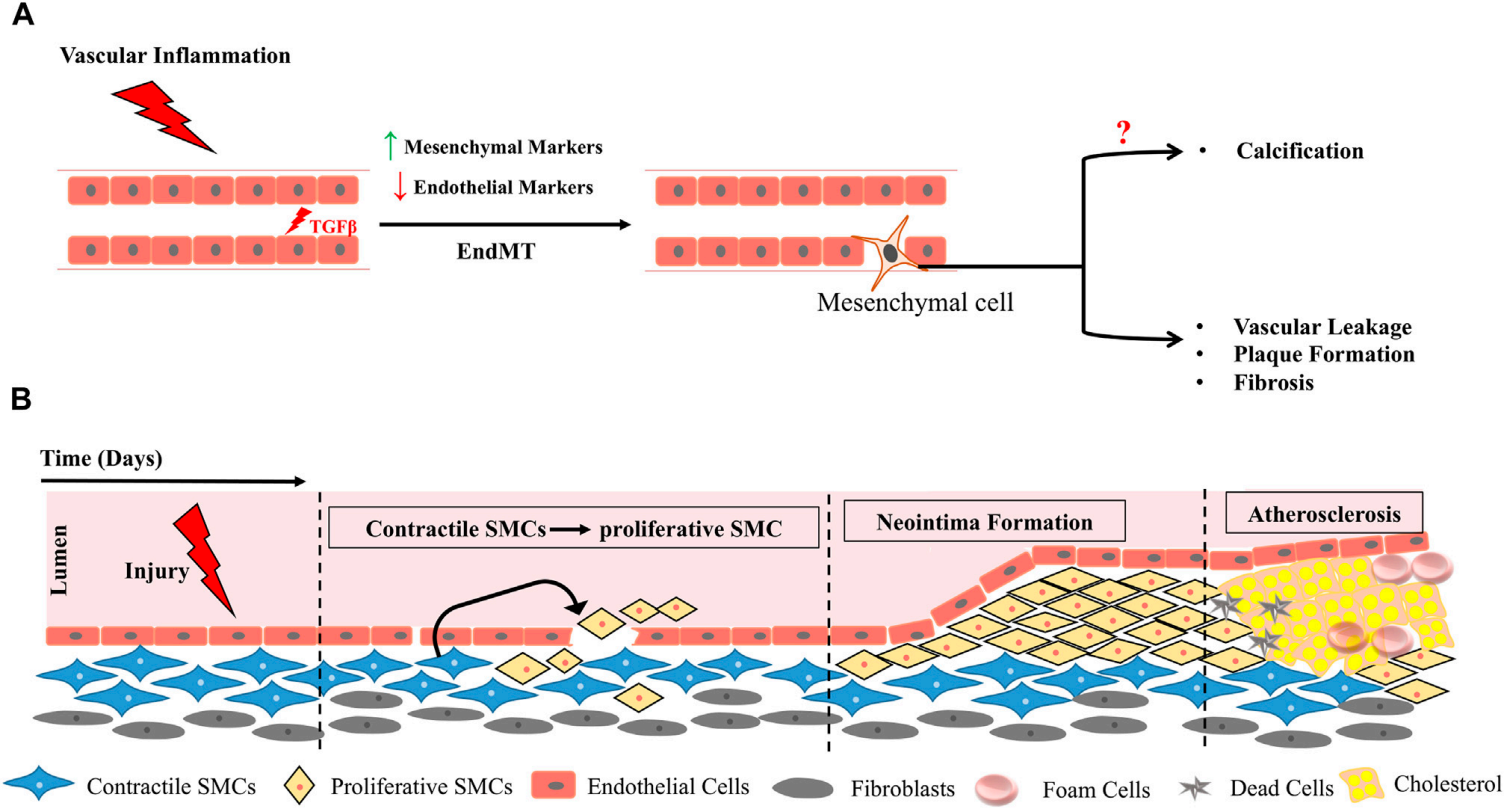
Vascular Inflammation triggers EndMT **(A)**, functional plasticity changes in smooth muscle cells (SMCs) and results in formation of neo-intima precipitating plaque deposition **(B)**.

Maintenance of cellular differentiation state is a requisite for maintenance of tissue integrity and tight epigenetic controls through development ascertains irrevocable fate transitions. This paradigm has been requisitioned through noble prize-winning studies wherein reprogramming of cellular states through nuclear transplantation and later induction of pluripotency by an embryonic transcription factor cocktail entailed pluripotent state to terminally differentiated fibroblasts. These ground breaking experiments prove that cells can be reinstructed to change their identity at least *in vitro*. Trans-differentiation is also now considered as a possibility where terminally differentiated cells can be instructed to convert to other differentiated cell types either directly or intermittent de-differentiation through a stem/progenitor state. Though most of these events were induced by *in vitro* manipulations, trans-differentiation as well as dedifferentiation of adult cells have been noted physiologically under injury, tissue inflammation and stress. In mammalian system, the best examples of injury/inflammation induced trans-differentiation are from the liver, pancreas, and the vascular systems (Figures 5A–D, 6).

By elegant lineage tracing of hepatocytes during injury, they were shown to initiate a bile duct epithelial cell like reprogramming upon ductal injuries in a process engaging Notch signaling (Yanger et al., 2013). The trans-differentiated cells were shown to exhibit morphological, structural, and molecular features of Biliary Epithelial Cells (BECs). In fact, severe biliary injury instigates hepatocytes to BEC transition (Michalopoulos et al., 2005). Chronic immune attack during biliary cirrhosis (wherein biliary cell’s proliferative capacity is compromised) instigates hepatocyte-BEC trans-differentiation to compensate the loss (Figure 5B). In a DDC model of liver injury, hepatocytes were shown to undergo ductal transition with intermittent expression of mesenchymal genes, these ducts undergo extensive expansion contribute to hepatic mass and convert back to differentiated hepatocytes upon cessation of injury (Figure 4A) indicating the importance of injury driven factors in driving intermittent/dynamic trans-differentiation events (Tarlow et al., 2014).

Cells within adult pancreas had been shown to exhibit endocrine plasticity physiologically in response to beta cell loss. An acute ablation of 99% of beta cell mass, (mimicking the Type 1 Diabetes, T1DM model), in adult mice had been shown to induce α cells to transdifferentiate into beta cells to compensate the loss and rescue the need for exogenous insulin for at least 6 months after β cell ablation (Thorel et al., 2010). A similar ablation of β cells in juvenile mice resulted in somatostatin producing δ cells to transdifferentiate into β cells (Chera et al., 2014). Thus in TIDM trans-differentiation could be a possible compensatory mechanism for beta cell loss (Figure 5D). Unlike T1DM, Type 2 Diabetes (T2DM) is due to insulin resistance developed over time and the resultant loss in beta cell function is due to chronic stress resulting in pancreatic inflammation (Eizirik et al., 2020). Surprisingly loss of beta cell to other endocrine cell types added to beta cell loss in T2DM in both murine model of diabetes as well as T2DM human subjects (Talchai et al., 2012; Guo et al., 2013). Loss of beta cell specific transcription factors such as MAFA/MAFB have been noted in human type 2 DM patients further emphasizing on loss of beta cell identity as one parameter contributing to diabetic pathology (Guo et al., 2013). Since chronic low grade inflammation and unresolved damage are pertinent features of the T2DM state it is possible that unresolved persistent inflammation could trigger these trans-differentiation events (Figure 5D). Chronic Pancreatitis is a typical inflammation triggered event characterized by fibrosis and acinar cell to ductal cell metaplasia. This is generally a reversible process resettling during injury resolution however if unresolved can end up in neoplastic lesions mostly in an oncogenic mutational background. Surprisingly in mouse models of chronic pancreatitis (without a background oncogenic mutation) induced by ER stress and marked by extensive inflammation, loss of acinar identity is marked by appearance of chemo-sensory tuft cells (Figure 5C). Trans-differentiated tuft cells show a strong inflammatory gene expression profile indicating that inflammation could have driven the tuft cell trans-differentiation. Lineage tracing of acinar cells indicate that these tuft cells arise from acinar through ductal metaplasia (DelGiorno et al., 2020). Further these Tuft cells are IL25 + which indicates a probable immunomodulatory roles in the inflamed pancreatic microenvironment. In human IgAnephropathy (IgAN) podocyte injury is marked by inflammasome activation as well as podocyte to macrophage trans-differentiation (Peng et al., 2019). All these evidences highlight the importance of chronic endogenous inflammatory triggers in initiating trans-differentiation events resulting in loss of tissue function.

A classic example of developmental trans-differentiation is emergence of HSCs directly from hemogenic endothelium (Figure 2C). Surprisingly a cell autonomous role of IFNγ-STAT3 signaling axis triggered by notch signaling and blood flow has been to shown to impact endothelial to hematopoietic conversion of HCC cells during HSC emergence (Sawamiphak et al., 2014). In adults, vascular inflammation had been shown to instigate Endothelial to mesenchymal transition (EndMT) in several pathological contexts including cerebral strokes, chronic renal failure, cardiovascular diseases and vascular calcifications. Brain diseases are commonly characterized by vascular fibrosis and cerebral stroke leads to endothelial cells undergoing EndMT which further progresses to vascular fibrosis thus impairing neurological function (Chen et al., 2020). Under conditions of chronic inflammation, neo-intima formation and endothelial dysfunction is facilitated through an EndMT transition triggered in most cases by induction of TGFβ pathway (Figure 6) (Pérez et al., 2017; Cho et al., 2018).

In fact EndMT serves as the link between inflammatory factors and plaque growth in atherosclerosis. In ApoE^−/−^ mice fed with western diet atherosclerotic lesions were observed in aortic roots with a concomitant increase in HDAC3. Even in human patients arteriosclerotic lesions in inflammatory areas were associated with HDAC3. HDAC3 inhibition alleviated EndMT by modulating inflammatory response indicating an intimate link between inflammatory triggers and EndMT in context of arteriosclerosis (Chen et al., 2021). Inflammation prone organs such as liver, pancreas, colon, and prostate are at high risk of cancer and unresolved chronic inflammation could be a risk factor for trans-differentiation events which when irrevocable could lead to advent of tumorigenesis. Thus, inflammation seems to be common trigger driving vascular loss due to EndMT.

## Conclusion and Future Perspective

Recent technical advancements in iPSC generation, PSC derived organoid cultures and gene editing have opened up new avenues in drug screening and disease modelling in a dish. However the potential clinical applications have not caught pace with the technical knowhow due to limitation of usage of these cells for *in vivo* therapeutic applications. Major bottlenecks include epigenetic instability, functional immaturity of the differentiated cells and subsequent immune rejection and engraftment upon transplantation. Further faithful mimicking of the ultrastructural and intricate functional components of endogenous tissues is a complicated task *in vitro*. Thus tissue niche based targeting approaches to aid endogenous repair and regeneration is an interesting strategy. Designing rational niche targeting strategies requires adequate knowledge of micro- and macro-environmental factors influencing stem cell decisions in their tissue habitats. Dynamic two-way interactions of stem cells with stromal components and resident or systemically recruited immune system players are pivotal in reshaping tissue response patterns to duress. Fetally patterned immune niches in most tissues are subjected to life-long sculpting in a demand driven process. In this premise inability of stem cells and/or immune niches to detect danger signals and eliminate or constructively adapt to crisis are central to aging, stem cell exhaustion and aberrant trans-differentiation events driving progressive degenerative disease. Thus breaking noxious iterative communication networks at stem cell-immune interfaces would be key for constructive regeneration. This would require identification of major molecular players at aberrant stem cell-immune interfaces to unravel novel immune-centric regenerative therapeutic possibilities which could also serve as biomarkers to detect early degeneration. Niche based targeting approaches would require a holistic understanding of tissue and organismal aging and degenerative processes. Since organismal aging is intimately associated with sterile bone marrow centric inflamm-aging, uncoupling these networks at the organismal levels in degenerative disease contexts could also be one possibility to reestablish tissue homeostasis and recovery. In addition, since innate immune system is ubiquitous in most organs finding unique components and interactors in tissue immune niches would be important to establish tissue directed targeting approaches in specific tissue pathologies without compromising overall immune fitness.
